# Synergistic Effect of Dual Electron-Cocatalysts for Enhanced Photocatalytic Activity: rGO as Electron-Transfer Mediator and Fe(III) as Oxygen-Reduction Active Site

**DOI:** 10.1038/srep13083

**Published:** 2015-08-14

**Authors:** Huogen Yu, Jing Tian, Feng Chen, Ping Wang, Xuefei Wang

**Affiliations:** 1 State Key Laboratory of Silicate Materials for Architectures, Wuhan University of Technology, Wuhan 430070, People’s Republic of China; 2 School of Chemistry, Chemical Engineering and Life Sciences, Wuhan University of Technology, Wuhan 430070, People’s Republic of China

## Abstract

For a high-performance cocatalyst-modified photocatalyst, an effective interfacial separation of photogenerated electron from its corresponding holes and its following reduction reaction at the active sites are highly required. However, it is difficult for a single-component cocatalyst to simultaneously realize the crucial functions. In this study, an effective interfacial transfer of photogenerated electrons and its following rapid oxygen-reduction can be easily realized in a dual electron-cocatalyst modified Fe(III)/rGO-TiO_2_ photocatalyst, where the rGO nanosheets function as an electron-transfer mediator for the effective transfer of photogenerated electrons from the TiO_2_ surface while the Fe(III) cocatalyst serves as an electron-reduction active site to promote the following interfacial oxygen reduction. In this case, the rGO nanosheets were firstly loaded on the TiO_2_ nanoparticle surface by a hydrothermal method and then the Fe(III) cocatalyst was further modified on the rGO nanosheets by an impregnation method to prepare the Fe(III)/rGO-TiO_2_ photocatalyst. It was found that the dual electron-cocatalyst modified Fe(III)/rGO-TiO_2_ photocatalyst showed an obviously higher photocatalytic performance than the naked TiO_2_ and single-cocatalyst modified photocatalysts (such as Fe(III)/TiO_2_ and rGO-TiO_2_) owing to the synergistic effect of rGO and Fe(III) bi-cocatalysts. The present work can provide some new insights for the smart design of high-efficiency photocatalytic materials.

Electron-cocatalyst modification on a photocatalyst surface has been widely demonstrated to be one of the most efficient strategies for the enhanced photocatalytic performance^1^,^2^,^3^,^4^,^5^,^6^,^7^. For a highly effective electron cocatalyst, several crucial functions should be carefully considered during photocatalytic reactions: (1) the electron cocatalyst firstly works as an electron sink to rapidly capture the photogenerated electron from a photo-excited photocatalyst, causing its effective separation with the photogenerated hole in the identical bulk material; (2) the electron cocatalyst then functions as an electron mediator to steadily transport the photogenerated electron from electron-captured sites to active reaction centers; (3) finally, the electron cocatalyst serves as the active reaction sites to effectively promote the interfacial reduction reaction (e.g. oxygen reduction for the decomposition of organic substances, while hydrogen production for the water splitting). As a consequence, it is very clear that a large amount of interfacial contacting sites with a strongly coupling interface between cocatalyst and photocatalytic materials, and the cocatalyst with a high electron mobility and rapid electron-reduction reaction should be highly required for a high-performance cocatalyst-modified photocatalysts^8^,^9^. Unfortunately, for a single-component cocatalyst, it is quite difficult to simultaneously realize the above vital functions. For examples, the interfacial contacting area between noble metal nanoparticles (such as Pt, Au, and Ag) and photocatalysts is very limited, while graphene/reduced graphene oxide (rGO) cocatalyst usually shows an obviously lower interfacial reduction rate than the well-known noble metal nanoparticles (Indeed, some researchers have attempted to load some noble metals as the active reaction sites on the graphene surface to improve the interfacial catalytic performance^10^,^11^). Therefore, it is highly desirable to further develop new strategy for the optimization of cocatalysts to improve the performance of photocatalytic materials. In addition, considering the expensive cost of various noble metal cocatalysts, it is quite interesting and worthwhile to explore new and cost-effective cocatalysts for the enhanced photocatalytic performance of cocatalyst-modified photocatalysts.

Graphene or rGO is a promising material in various potential applications due to its unique charge carrier mobility properties, large specific surface area and chemical stability^12^,^13^,^14^,^15^,^16^. In photocatalytic field, it has been widely demonstrated that the photocatalytic performance of various photocatalysts can be greatly improved by the addition of graphene nanosheets^17^,^18^,^19^,^20^,^21^,^22^,^23^. The principal reason is that the graphene or rGO serves as an electron mediator to accelerate the separation efficiency of photogenerated electrons and holes due to its excellent electron mobility. In our recent results, we also found that the rGO nanosheets could be loaded on the TiO_2_ surface with a strongly coupling interface by a hydrothermal method, causing an obvious enhancement of its photocatalytic activity^24^,^25^. In addition, the transition metal Fe(III) and Cu(II) has been widely demonstrated to be a highly efficient electron cocatalyst for the interfacial oxygen reduction during photocatalytic decomposition of organic pollutants^26^,^27^,^28^,^29^,^30^,^31^,^32^,^33^,^34^,^35^. Considering their remarkably different advantages of rGO nanosheets and Fe(III) cocatalyst, it is expected that the photocatalytic performance of TiO_2_ can be further improved by the simultaneous modification of rGO nanosheets and Fe(III) cocatalysts.

In this study, a dual electron-cocatalyst modified TiO_2_ photocatalyst (Fe(III)/rGO-TiO_2_) with a high photocatalytic performance was developed by a facile two-step route including the initial synthesis of rGO-TiO_2_ composite and the following surface loading of Fe(III) cocatalyst on the rGO nanosheets. In this case of Fe(III)/rGO-TiO_2_ photocatalyst, the dual electron cocatalysts of rGO and Fe(III) exhibit two different functions during photocatalytic reaction, namely, the rGO nanosheets as an electron mediator cause the steady capture and rapid transportation of photogenerated electrons from the conduction band (CB) of TiO_2_, while the Fe(III) serves as an effective oxygen-reduction active site for the following interfacial reduction reaction of oxygen. In view of a high specific surface area of rGO nanosheets, the Fe(III) cocatalyst was easily loaded on the rGO surface in the rGO-TiO_2_ composite. On the basis of the experimental results, a synergistic effect mechanism of rGO and Fe(III) bi-cocatalysts was proposed to account for its improved photocatalytic performance. To the best of our knowledge, this is the first report about the enhanced photocatalytic performance of TiO_2_ by a dual electron-cocatalyst modification of rGO nanosheets and Fe(III) ions. Considering a large-scale production of rGO from the graphite with a low cost, the abundant Fe and C elements in natural resources, and the facile and green synthetic route, the resulting Fe(III)/rGO-TiO_2_ photocatalyst can be regarded as one of the most promising photocatalytic materials for the various potential applications. In addition, this work can provide some new insights for the smart design and preparation of inexpensive and high-efficiency photocatalytic materials.

## Results

**Figure 1:**
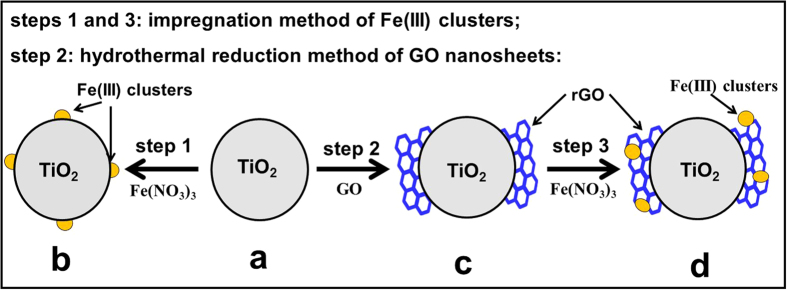
Schematic diagram illustrating the controllable preparation of the samples: (**a**) TiO_2_; (**b**) Fe(III)/TiO_2_; (**c**) rGO-TiO_2_; (**d**) Fe(III)/rGO-TiO_2_.

Various cocatalyst-modified photocatalysts such as TiO_2_, Fe(III)/TiO_2_, rGO-TiO_2_ and Fe(III)/rGO-TiO_2_ can be easily prepared by facile solution routes (Fig. 1). Firstly, rutile TiO_2_ precursor (Fig. 1a) was obtained from P25 TiO_2_ nanoparticles after high-temperature calcination which can produce a very clear and hydrophilic TiO_2_ surface for the following rapid coupling with GO nanosheets. As a consequence, when the calcined TiO_2_ particles were dispersed into the GO solution, a homogeneous and stable GO-TiO_2_ suspension with a strongly coupling interface was formed immediately. After hydrothermal reduction of GO to rGO, the strongly coupling interface can be well maintained in the resultant rGO-TiO_2_ composite (Fig. 1c), as shown in our recent results^24^. In fact, the GO nanosheets were *in situ* reduced to rGO on the TiO_2_ surface via a hydrothermal deoxygenation/dehydration reaction. For the surface modification of Fe(III) cocatalyst on the TiO_2_ and rGO-TiO_2_ photocatalysts (Fig. 1b,d), a well-known impregnation method of Fe(III) clusters was performed at 80 ^°^C with a mild condition. Considering a low-temperature loading route for the Fe(III) cocatalyst, it is quite believed that the Fe(III) cocatalyst is only loaded on the photocatalyst surface, but not doped into the lattices of TiO_2_ and rGO nanosheets.

**Figure 2:**
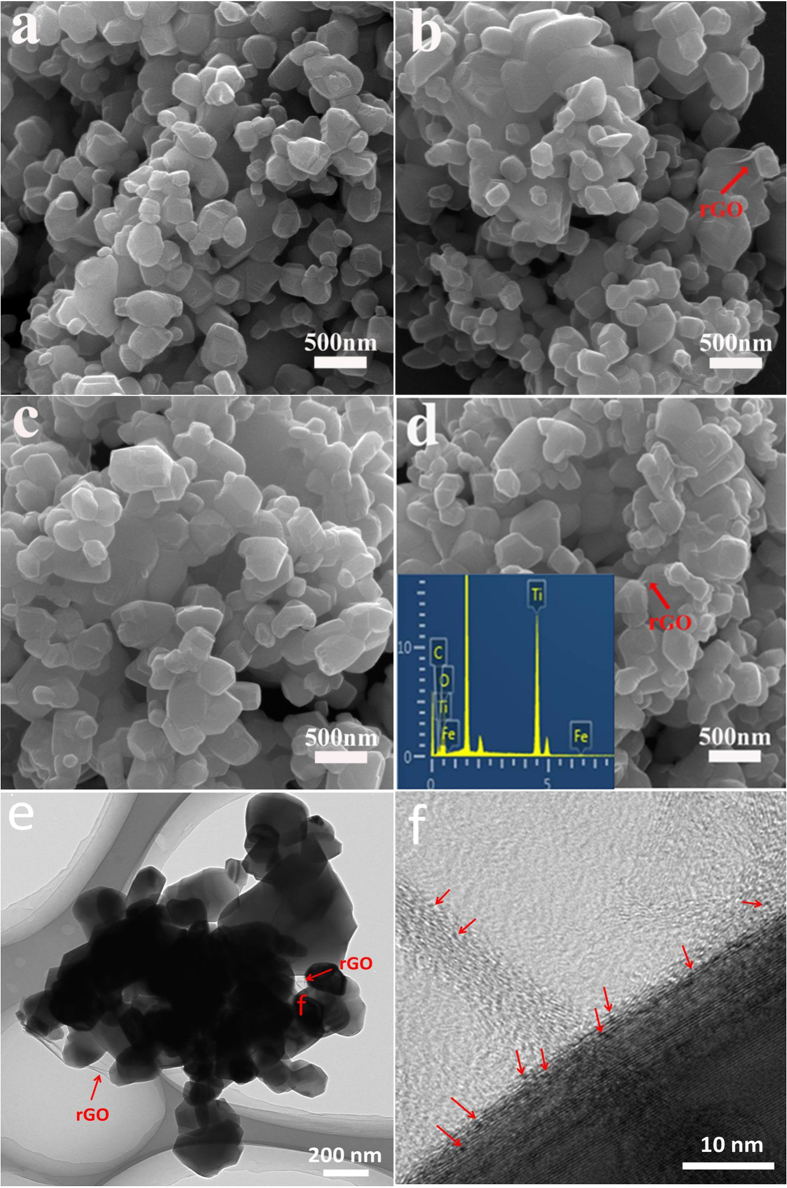
FESEM images of (**a**) TiO_2_; (**b**) rGO-TiO_2_; (**c**) Fe(III)/TiO_2_; (**d**) Fe(III)/rGO-TiO_2_. TEM (**e**) and HRTEM (**f**) images of Fe(III)/rGO-TiO_2_ photocatalyst: the red arrows in (**e**) and (**f**) showing the rGO and Fe(III) clusters, respectively.

The controlled preparation of various cocatalyst-modified TiO_2_ photocatalysts can firstly be demonstrated by FESEM, TEM and XRD results. Figure 2 shows the FESEM images of the TiO_2_, rGO-TiO_2_, Fe(III)/TiO_2_, and Fe(III)/rGO-TiO_2_. It can be seen that the TiO_2_ sample is composed of irregular particles with a size range of 100–600 nm (Fig. 2a), suggesting an obvious growth of the TiO_2_ particles during high-temperature calcination (P25 nanoparticles with a size of 20–50 nm). The corresponding XRD result suggests the successful phase transformation of rutile-anatase mixing phase to pure rutile (Figure S1). After the rutile TiO_2_ surface is modified with Fe(III) cocatalyst, the resulting Fe(III)/TiO_2_ (Fig. 2c) shows a similar morphology as the TiO_2_ sample due to a very low amount of Fe(III) cocatalyst (Fe/TiO_2 _= 0.05 wt%). As for the rGO-TiO_2_ (Fig. 2b) and Fe(III)/rGO-TiO_2_ (Fig. 2d) samples, they also show very similar particle morphologies with the pure TiO_2_. In addition, it was found that the rGO nanosheets were tightly covered on the TiO_2_ particle surface (shown in the arrow), indicating a well interfacial interaction of rGO nanosheets with the TiO_2_ nanoparticles, in good agreement with our strategy (Fig. 1). The corresponding TEM images of Fe(III)/rGO-TiO_2_ further demonstrated the well coupling of rGO with the TiO_2_ particles, as shown in Fig. 2e,f. Moreover, the Fe(III) clusters with a very small size (less than 1 nm) are homogeneously dispersed on the surface of rGO nanosheets (showed by the red arrows in Fig. 2f), suggesting that the impregnation method is an excellent strategy for the loading of Fe(III) cocatalyst. In addition, on the basis of XRD results (Figure S1), it is found that all the cocatalyst-modified TiO_2_ photocatalysts show a comparable diffraction-peak intensity and full width at half-maximum compared with the pure TiO_2_ sample, revealing that the crystallization and crystallite size of TiO_2_ photocatalyst are not affected by the cocatalyst modification processes owing to their mild conditions. According to the EDX result, the Fe element in the Fe(III)/rGO-TiO_2_ composite (Fig. 2d) is calculated to be 0.1 wt%. However, it should be noted that the Fe(III) clusters and its corresponding diffraction peaks cannot be observed in the FESEM image and XRD pattern, respectively, owing to its very limited amount. Considering the well coupling interface of rGO nanosheets and TiO_2_ nanoparticles (Fig. 2b,d), it is expected that the photogenerated electrons can be steadily transferred from TiO_2_ particles to the rGO nanosheets.

**Figure 3:**
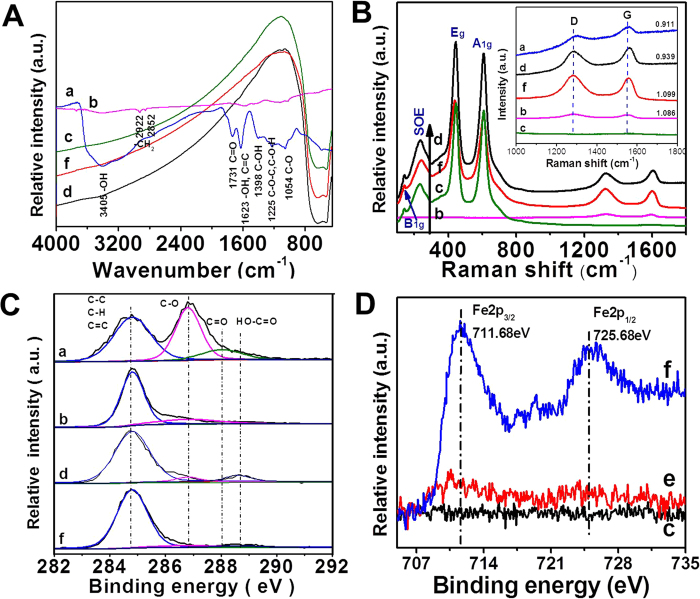
(**A**) FTIR spectra, (**B**) Raman spectra, (**C,D**) XPS spectra of C 1 s (**C**) and Fe 2p (**D**) of various samples: (**a**) GO; (**b**) rGO; (**c**) TiO_2_; (d) rGO-TiO_2_; (**e**) Fe(III)/TiO_2_; (**f**) Fe(III)/rGO-TiO_2_.

FTIR and Raman spectra can further demonstrate the formation of Fe(III)/rGO-TiO_2_ photocatalyst. Figure 3A shows the FTIR spectra of various samples. It is clear that the GO shows many strong absorption peaks corresponding to various oxygen functional groups (C ‖ O, C—OH, C—O—C, C—O—H and C—O)^10^,^36^. After hydrothermal treatment of the GO, the intensity of all absorption peaks corresponding to oxygen-containing groups has a significant decrease, demonstrating that the hydrothermal treatment is an effective strategy for the successful reduction of GO to rGO. For the rutile TiO_2_ nanoparticles, the wide absorption peak at 400–900 cm^−1^ is attributed to the stretching vibration of Ti—O—Ti bonds in crystalline TiO_2_^37^, while the corresponding absorption in the rGO-TiO_2_ and Fe(III)/rGO-TiO_2_ composites can be attributed to the stretching vibration of Ti—O—Ti and the possible Ti—O—C bonds formed during hydrothermal reaction. Figure 3B shows the Raman spectra of various samples. It is clear that rutile TiO_2_ shows strong Raman characteristic peaks at 145 cm^−1^ (B_1g_), 448 cm^−1^ (E_g_), 613 cm^−1^ (A_1g_) and 238 cm^−1^ for second order effect^38^. In addition, the Raman spectra in the inset of Fig. 3B show the characteristic D band at 1347 cm^–1^ and G band at 1604 cm^–1^ in the GO, rGO and rGO-modified TiO_2_ photocatalysts. It is well reported that the intensity ratio of the D band to the G band usually measures the defects/disorders in GO or graphene, and a small intensity ratio of I_D_/I_G_ can be assigned to less sp^3^ defects/disorders and larger average size of the in-plane graphitic crystallite sp^2^ domains^36^,^39^. Compared with the GO (0.911), the Fe(III)/rGO-TiO_2_ composite shows a higher *I*_D_/*I*_G_ ratio (1.099), indicating that the rGO in Fe(III)/rGO-TiO_2_ composite contains more sp^3^ defects. The possible reason for the increased sp^3^ defects can be attributed to the formation of strong interfacial interaction (such as the Ti—O—C bond) between the TiO_2_ nanoparticles and rGO nanosheets^39^.

XPS technology can provide further information about the formation of Fe(III)/rGO-TiO_2_ photocatalyst, as shown in Fig. 3C, D. After hydrothermal reaction of GO nanosheets, the chemical state of carbon atoms in the hexagonal lattice of graphene was changed significantly owing to the deoxygenation reaction, which can be well illustrated in Fig. 3C. The C1s XPS spectrum of the GO nanosheets clearly shows the presence of four types of carbon bonds, namely, the non-oxygenated ring carbons including C—C, C—H, and C‖C (284.8 eV), the C—O groups (286.8 eV), the carbonyl carbon in C‖O (288.0 eV) and the carboxylate carbon in O‖C—OH (288.6 eV). After hydrothermal treatment of the GO, the XPS peak intensity of these carbon–oxygen species in the resultant rGO shows an obvious decrease, suggesting the effective reduction of GO^40^,^41^. Considering an identical hydrothermal condition with the rGO nanosheets, it is quite reasonable that the rGO in rGO-TiO_2_ and Fe(III)/rGO-TiO_2_ system shows a similar surface microstructures. Figure 3D shows the high-resolution XPS spectra of Fe 2p. Compared with the pure TiO_2_ samples, new XPS peaks of Fe element at ca. 711.5 eV, which corresponds to the binding energy of Fe 2p_3/2_ for the ferric ion^27^,^42^, are found in the Fe(III)/TiO_2_ and Fe(III)/rGO-TiO_2_ samples. According to the element component analysis based on the XPS results (Table 1), it is found that the amount of Fe(III) cocatalyst in the Fe(III)/TiO_2_ and Fe(III)/rGO-TiO_2_ photocatalysts is about 1.17 and 5.38 at.%, respectively. However, according to the atomic absorption spectrometry analysis, the Fe amount in Fe(III)/rGO-TiO_2_ was about 0.44 wt%, which suggests that most of the Fe element has been loaded on the surface of graphene nanosheets in rGO-TiO_2_ composite. Therefore, the above experimental results strongly suggested that the rGO and Fe(III) cocatalyst has successfully been modified on the surface of TiO_2_ photocatalyst.

**Figure 4:**
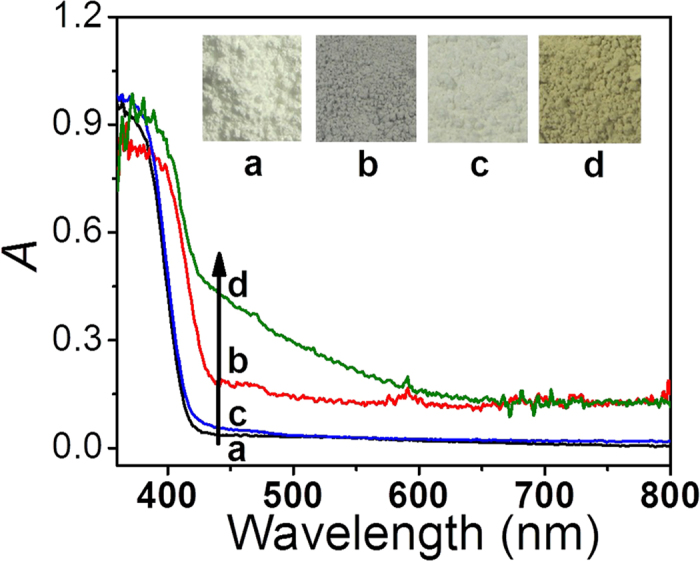
UV-vis spectra and the corresponding photographs (inset): (**a**) TiO_2_; (**b**) rGO-TiO_2_; (**c**) Fe(III)/TiO_2_; (**d**) Fe(III)/rGO-TiO_2_.

Figure 4 shows the UV-vis spectra of TiO_2_, rGO-TiO_2_, Fe(III)/TiO_2_ and Fe(III)/rGO-TiO_2_ samples. It is clear that the Fe(III)/TiO_2_ shows a similar UV-vis spectrum as the pure TiO_2_ owing to a very limited Fe(III) cocatalyst (Fig. 4a,c). After the rGO was loaded on the TiO_2_ surface, the absorption edge of rGO-TiO_2_ composite (Fig. 4b) shows a slight red-shift to a higher wavelength, and an enhanced visible-light absorption in the range of 400–800 nm can be observed owing to the presence of black rGO nanosheets. When both of the rGO nanosheets and Fe(III) cocatalyst are simultaneously deposited on the TiO_2_ surface to form Fe(III)/rGO-TiO_2_ photocatalyst (Fig. 4d), it is found that the visible-light absorption in the range of 400–600 nm can be further improved. The enhanced absorption can be attributed to the d-d transition of Fe(III) cocatalyst, clearly revealing a higher amount of Fe(III) cocatalyst in the Fe(III)/rGO-TiO_2_ than that in the Fe(III)/TiO_2_ (Fig. 4c) owing to the presence of rGO nanosheets, in good agreement with the XPS results. In fact, the colour change from grey colour of rGO-TiO_2_ to greyish yellow of Fe(III)/rGO-TiO_2_ can further provide the strong evidence for the controlled synthesis of Fe(III)/rGO-TiO_2_, as shown in the inset of Fig. 4.

## Discussion

**Figure 5:**
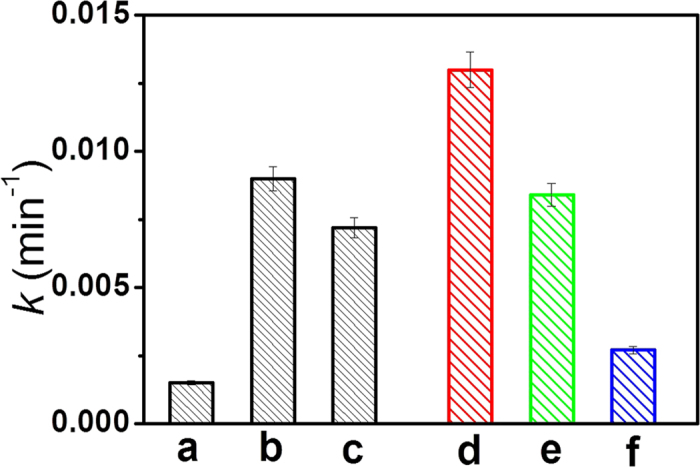
Rate constant (*k*) of MO decomposition by various photocatalysts: (**a**) TiO_2_; (**b**) rGO-TiO_2_; (**c**) Fe(III)/TiO_2_; (**d**) Fe(III)/rGO-TiO_2_; (**e**) rGO-TiO_2_ obtained from the sample (**d**) after the removal of Fe(III) by HCl solution; (**f**) TiO_2_ obtained from the sample (**e**) after the removal of rGO by high-temperature calcination.

The photocatalytic performances of TiO_2_, rGO-TiO_2_, Fe(III)/TiO_2_ and Fe(III)/rGO-TiO_2_ samples were first evaluated by photocatalytic decolorization of MO aqueous solution (Fig. 5). In the dark, no change in the concentration of MO was observed in the presence of different photocatalysts. Furthermore, visible-light illumination in the absence of photocatalysts did not result in the photocatalytic decolorization of MO. Figure 5 shows the corresponding photocatalytic rate constant *k* of different photocatalysts. For rutile TiO_2_, it exhibits a relative low photocatalytic activity and the *k* value is about 0.002 min^−1^. When the Fe(III) and rGO cocatalysts are grafted on the TiO_2_ surface, respectively, both of the resultant Fe(III)/TiO_2_ and rGO-TiO_2_ show an obviously improved photocatalytic performance with a *k* value of 0.007 and 0.009 min^−1^, respectively. More specifically, the Fe(III)/rGO-TiO_2_ photocatalyst possesses the highest photocatalytic activity (*k *= 0.013 min^−1^). In fact, the amount of Fe(III) on the rGO surface has a great effect on the photocatalytic performance of Fe(III)/rGO-TiO_2_ photocatalyst, and the optimized amount of Fe(III) cocatalyst was about 0.5 wt% (Figure S2), which is obviously higher than that in the Fe(III)/TiO_2_ photocatalyst (0.05 wt%). To further investigate the photocatalytic ability of cocatalyst-modified TiO_2_, the dimethyl phthalate (DMP) solution was also used to evaluate their photocatalytic performance. It is found that the Fe(III)/rGO-TiO_2_ photocatalyst can maintain a stable and efficient photocatalytic performance (Figure S3).

It is very interesting and worthwhile to investigate the potential photocatalytic mechanism of Fe(III)/rGO-TiO_2_ photocatalyst. The rutile TiO_2_ photocatalyst exhibits obvious photocatalytic activity for the decomposition of organic pollutions owing to its strong oxidation power of photogenerated holes (+2.63 V, vs. SHE, pH = 7) and suitable reduction potential of photogenerated electrons (−0.37 V, vs. SHE, pH = 7) (Figure S4a)^43^,^44^. However, the photogenerated electrons and holes are easily recombined in the single-component rutile TiO_2_, resulting a low photocatalytic efficiency. When the Fe(III) ions are grafted onto the TiO_2_ surface to form Fe(III)/TiO_2_ photocatalyst, the photogenerated electrons on the TiO_2_ CB can transfer to the Fe(III) cocatalyst to form Fe(II) owing to its more positive potential of Fe^3+^/Fe^2+^ (0.771 V, vs. SHE)^45^ than the CB of TiO_2_, which can promote the separation efficiency of photo-generated electrons and holes in bulk TiO_2_ (Figure S4c). More importantly, the as-formed Fe(II) cocatalyst can reduce oxygen rapidly by a possible multi-electron reduction mechanism (4Fe^2+ ^+ O_2 _+ 4H^+^ → 4Fe^3+^ + 2H_2_O or 4Fe^2+ ^+ O_2 _+ 2H_2_O → 4Fe^3+ ^+ 2OH^−^)^45^, causing a significant enhancement of photocatalytic performance. Compared with the Fe(III)/TiO_2_ photocatalyst, however, the rGO-TiO_2_ shows a completely different improved mechanism (Figure S4b). In this case, the well coupling interface and a large number of interfacial contacting sites between the rGO nanosheets and TiO_2_ particles provide an excellent platform for the rapid and efficient transfer of photogenerated electrons from the TiO_2_ CB. As a consequence, the rGO nanosheets work as an effective electron mediator for the rapid capture and steady transportation of photogenerated electrons from the TiO_2_ surface, resulting in a lower recombination rate of photogenerated charges in the bulk TiO_2_ and a higher photocatalytic activity.

**Figure 6:**
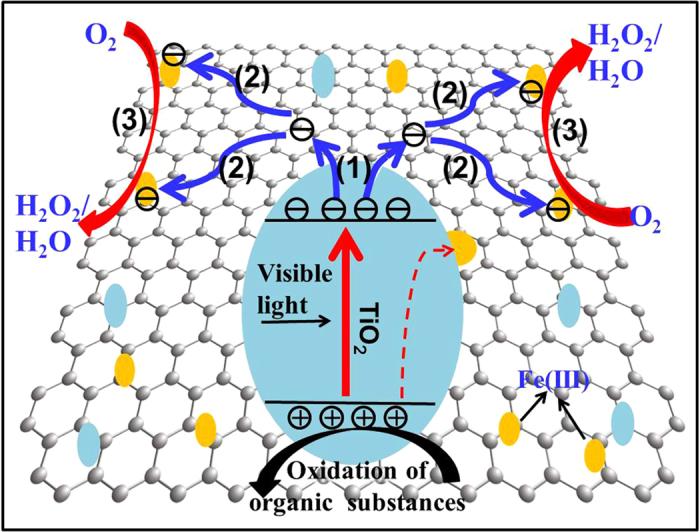
Schematic diagram illustrating the enhanced photocatalytic performance of Fe(III)/rGO-TiO_2_ photocatalyst. Step (1): rGO as an electron sink to capture the photogenerated electrons from the TiO_2_ surface; Step (2): rGO as an electron mediator to transfer electrons from the capture sites to the Fe(III) clusters; Step 3: Fe(III) clusters as the oxygen-reduction active sites for the rapid reduction of oxygen.

When both of the rGO nanosheets and Fe(III) cocatalyst are simultaneously deposited on the surface of TiO_2_, the further improved photocatalytic performance of Fe(III)/rGO-TiO_2_ photocatalyst can be well explained by their synergistic effect, as shown in Figure S4d and Fig. 6. In the presence of rGO, the photogenerated electrons of TiO_2_ can first transfer to the capture sites of rGO nanosheets (Step (1) in Fig. 6). In this case, the rGO works as an electron sink to rapidly capture the photogenerated electron from the TiO_2_ surface, while the photogenerated holes are still remained on the TiO_2_ bulk to oxidize the organic substances, resulting in an effective separation of photogenerated charges. Considering a large amount of interfacial contacting sites with a strongly coupling interface between rGO nanosheets and TiO_2_ nanoparticles (Figs 1 and 2), it can be deduced that the rGO has a higher separation rate for the photogenerated charges than the well-known nanoparticle-like cocatalyst such as the Fe(III) cocatalyst in the Fe(III)/TiO_2_. After the capture of photogenerated electrons, the rGO nanosheets then function as an electron mediator to transport the captured electrons to the oxygen-reduction active sites owing to its excellent carrier mobility property (Step (2) in Fig. 6). Finally, considering that rGO nanosheets can serve as an excellent scaffold to homogeneously anchor the Fe(III) cocatalyst, the highly dispersed Fe(III) clusters serve as the effective oxygen-reduction active sites for the highly efficient reduction of oxygen (Step (3) in Fig. 6). Thus, it is very clear that the excellent synergetic effect between Fe(III) and rGO cocatalysts greatly promote the effective separation of photogenerated charges, the steady transportation of photogenerated electrons and its following oxygen reduction, resulting in the improved photocatalytic performance of Fe(III)/rGO-TiO_2_ composites. In fact, in addition to the rutile TiO_2_, the synergetic effect between Fe(III) and rGO cocatalysts can also be demonstrated in the well-known P25 nanoparticles (a *S*_BET_ of ca. 50 m^2^/g, 80% anatase and 20% rutile) under UV-light irradiation (Figure S5), where the UV-light photocatalytic performance of Fe(III)/rGO-P25 is obviously higher than that of rGO-P25 and P25.

**Figure 7:**
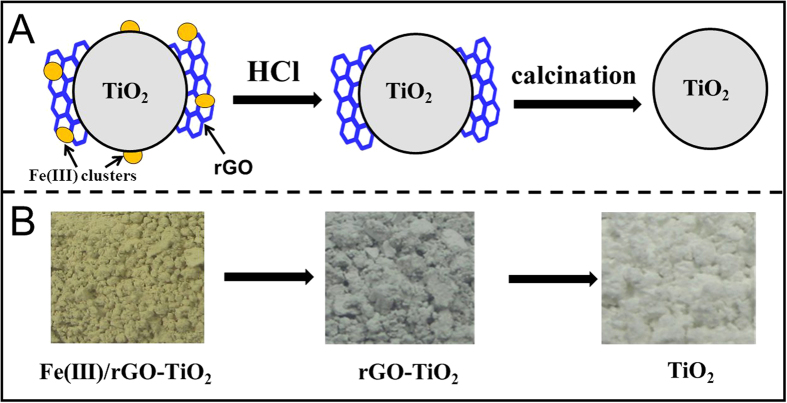
(**A**) Schematic diagram illustrating the gradual removal of Fe(III) and rGO from the TiO_2_ surface; (**B**) the corresponding photographs of the resultant samples.

To further demonstrate the synergistic effect of Fe(III) and rGO cocatalysts for the enhanced photocatalytic performance of Fe(III)/rGO-TiO_2_, a series of controlled experiments are performed (Fig. 7A). Firstly, Fe(III) cocatalyst on the surface of Fe(III)/rGO-TiO_2_ photocatalyst are selectively removed by HCl solution (0.1 mol/L), which can be clearly demonstrated by its colour change from greyish yellow to grey (Fig. 7B). In this case, the corresponding *k* value of the resultant rGO-TiO_2_ sample decreases significantly from 0.013 to 0.008 min^−1^ (Fig. 5e), a value comparable to that of the as-prepared rGO-TiO_2_ photocatalyst (Fig. 5b). With further selective removal of rGO by high-temperature calcination in the air (500 ^°^C), the resulting TiO_2_ sample shows a further decreased decomposition performance (k = 0.003 min^−1^) (Fig. 5f) and its colour returns to white completely (Fig. 7B). Therefore, the above controlled experimental results strongly support that the synergistic effect of Fe(III) and rGO bi-cocatalysts is the main reason for the enhanced photocatalytic activity of Fe(III)/rGO-TiO_2_.

In summary, a new dual electron-cocatalyst modified Fe(III)/rGO-TiO_2_ photocatalyst was successfully prepared by a facile two-step route including the initial synthesis of rGO-TiO_2_ composite and the following surface loading of Fe(III) cocatalyst on the rGO nanosheets. It was found that the Fe(III)/rGO-TiO_2_ photocatalyst showed an obviously higher photocatalytic performance than the naked TiO_2_ and single-cocatalyst modified photocatalysts (such as Fe(III)/TiO_2_ and rGO-TiO_2_). On the basis of the experimental results, a synergistic effect mechanism of rGO and Fe(III) bi-cocatalysts was proposed to account for its improved photocatalytic performance, namely, the rGO nanosheets function as an electron-transfer mediator to rapidly capture and transfer the photogenerated electron from TiO_2_ surface, while the Fe(III) cocatalyst serves as an effectively active site for the following oxygen reduction. The present work will provide new insight for the smart design of new cocatalyst-modified photocatalytic materials with a high performance.

## Methods

### Chemicals

Commercial Degussa TiO_2_ nanoparticles (P25 TiO_2_, a *S*_BET_ of ca. 50 m^2^/g, 80% anatase and 20% rutile) was pre-treated at 900 ^o^C for 3 h, and the resultant rutile TiO_2_ was used as the TiO_2_ precursor in this study. Graphene oxide (GO) was synthesized from natural graphite powder (99.95%) by a modified Hummer’s method shown in our previous studies, and the brown GO (0.2 wt%) solution was obtained by ultrasonic dispersion of GO (0.5 g) in deionized water (250 mL) for 1 h. All the other reagents (analytical grade) were supplied by Shanghai Chemical Ltd. (P.R. China) and used as received without further purification.

### Preparation of rGO-TiO_2_ composite

The rGO-TiO_2_ composite was obtained via one-step hydrothermal method. According to our results, for the rGO-TiO_2_ photocatalyst by using rutile TiO_2_ as the precursor, it was found that when the rGO amount (rGO/TiO_2_) was 0.1 wt%, the prepared rGO-TiO_2_ showed the highest photocatalytic performance. Therefore, in this case, the amount of rGO in the rGO-TiO_2_ composite was controlled to be 0.1 wt%. For a typical synthesis, 1.6 g of TiO_2_ was first dispersed into 15 mL deionized water, and then 0.8 mL of GO solution was added into the above mixing solution. After strong stirring for 2 h, the resultant homogeneous suspension was transformed into Teflon-sealed autoclave and then maintained at 160 ^o^C for 5 h. After being cooled to room temperature naturally, the resulting composite was washed with distilled water for several times, and dried in vacuum at 60 ^o^C for 8 h to obtain the rGO-TiO_2_ composites. For comparison, the GO solution was also hydrothermally treated under an identical condition, and the resulting black sample was referred to as rGO.

### Preparation of Fe(III)/TiO_2_

The Fe(III)/TiO_2_ photocatalyst was synthesized by an impregnation technique similar to our previous studies. According to our experimental results, it was found that when the amount of Fe(III) cocatalyst (compared to TiO_2_) was controlled to be 0.05 wt%, the resultant Fe(III)/TiO_2_ showed the highest photocatalytic activity. Therefore, in this study, the Fe(III)/TiO_2_ (Fe(III)/TiO_2 _= 0.05 wt%) was used as the reference sample and was referred to be Fe(III)/TiO_2_. For a typical synthesis, 0.4 g of TiO_2_ was dispersed into 10 mL of Fe(NO_3_)_3_ solution (0.36 μmol/L, pH = 2) under stirring and was then maintained at 80 ^o^C for 1 h. The resulting composite was filtrated, rinsed with distilled water, and dried at 60 ^o^C for 8 h to obtain the Fe(III)/TiO_2_ sample.

### Preparation of Fe(III)/rGO-TiO_2_

The Fe(III)/rGO-TiO_2_ photocatalyst was prepared under an identical experimental condition with the Fe(III)/TiO_2_ photocatalyst by using rGO-TiO_2_ as the precursor. In addition, the amount of Fe(III) in the Fe(III)/rGO-TiO_2_ composites was controlled to be 0.05, 0.1, 0.2, 0.5 and 1.0 wt%, and the resulting sample can be referred to as Fe(III)/rGO-TiO_2_ (*X*), where *X* represents 0.05, 0.1, 0.2, 0.5, and 1.0, respectively. According to the results by atomic absorption spectrometry, when the amount of Fe(III) precursor was controlled to be 0.05, 0.1, 0.2, 0.5 and 1.0 wt%, and the real amount of Fe(III) in the resultant Fe(III)/rGO-TiO_2_ composites was 0.048, 0.093, 0.14, 0.33 and 0.69 wt%, respectively. However, as showed in this study (Figure S2), when the *X* was 0.5, the prepared Fe(III)/rGO-TiO_2_ photocatalyst showed the highest photocatalytic performance. Thus, the Fe(III)/rGO-TiO_2_ (0.5) was used as the reference sample and was referred to be Fe(III)/rGO-TiO_2_ in this work.

### The selective removal of Fe(III) and rGO

For the selective removal of Fe(III) and rGO cocatalysts from the TiO_2_ surface, the Fe(III)/rGO-TiO_2_ sample was firstly dispersed into a HCl solution (0.1 mol/L) and then was calcined at 500 ^o^C for 2 h in the air.

### Characterization

X-ray diffraction (XRD) patterns were obtained on a D/MAXRBX-ray diffractometer (Rigaku, Japan). Morphological analysis was performed with an S-4800 field emission scanning electron microscope (FESEM) (Hitachi, Japan) with an acceleration voltage of 10 kV. TEM/HRTEM image was conducted using a JEM-2100F transmitting electron microscope. Raman spectra were collected using an INVIA spectrophotometer (Renishaw, UK). Fourier Transform Infrared spectra (FTIR) were acquired using a Nexus FT-IR spectrophotometer (Thermo Nicolet, America). X-ray photoelectron spectroscopy (XPS) measurements were done on a KRATOA XSAM800 XPS system with Mg Kα source. All the binding energies were referenced to the C1 s peak at 284.8 eV for the surface adventitious carbon. The amount of Fe(III) in Fe(III)/rGO-TiO_2_ was performed on an atomic absorption spectrometry (GBC AVANTA-M, Australia). UV–vis absorption spectra were obtained using a UV–visible spectrophotometer (UV-1240, SHIMADZU, Japan).

### Photocatalytic activity

The evaluation of photocatalytic activity of the prepared samples for the photocatalytic decomposition of methyl orange (MO) and dimethyl phthalate (DMP) solutions was evaluated at ambient temperature. Experimental details were shown as follows: 0.1 g of the sample was dispersed into 10 mL of MO solution (20 mg/L) or DMP solution (10 mg/L) in a disk with a diameter of *ca.* 5 cm. The solution was allowed to reach an adsorption-desorption equilibrium among the photocatalyst, MO (or DMP) and water before irradiation. A 350-W xenon lamp equipped with a UV-cutoff filter (providing visible light > 400 nm wavelength) was used as a visible-light source. The average light intensity striking the liquid surface of the reaction solution was about 40 mW/cm^2^. The concentration of the MO (or DMP) was determined by an UV-visible spectrophotometer (UV-1240, SHIMADZU, Japan). As for the MO (or DMP) aqueous solution with low concentration, its photocatalytic decolorization is a pseudo-first-order reaction and its kinetics may be expressed as ln(*c*/*c*_0_) = −*kt*, where *k* is the apparent rate constant, and *c*_0_ and *c* are the MO (or DMP) concentrations at initial state and after irradiation for *t* min, respectively. For the repeated photocatalytic performance, the photocatalysts were first separated by centrifugation, washed with distilled water, and were then re-dispersed into the MO (or DMP) solutions.

## Additional Information

**How to cite this article**: Yu, H. *et al.* Synergistic Effect of Dual Electron-Cocatalysts for Enhanced Photocatalytic Activity: rGO as Electron-Transfer Mediator and Fe(III) as Oxygen-Reduction Active Site. *Sci. Rep.* **5**, 13083; doi: 10.1038/srep13083 (2015).

## Supplementary Material

Supplementary Information

## Figures and Tables

**Table 1:** Composition (at.%) of the various samples according to XPS analysis.

**Samples**	**C**	**O**	**Ti**	**Fe**
GO	65.48	34.52	0	0
rGO	85.84	14.16	0	0
TiO_2_	39.21	41.56	19.23	0
rGO/TiO_2_	49.67	38.26	12.07	0
Fe(III)/TiO_2_	41.68	41.93	15.22	1.17
Fe(III)/rGO-TiO_2_	40.47	42.07	12.08	5.38
