# Supplementary material for: Synergistic Effect of Dual Electron-Cocatalysts for Enhanced Photocatalytic Activity: rGO as Electron-Transfer Mediator and Fe(III) as Oxygen-Reduction Active Site
Source: Sci Rep. 2015 Aug 14;5:13083. doi: 10.1038/srep13083 (PMC4536495; doi:10.1038/srep13083)
Supplement: Supplementary Information [file srep13083-s1.doc]

Supplementary

**Synergistic Effect of Dual electron-cocatalysts for Enhanced Photocatalytic Activity: rGO as Electron-Transfer Mediator and Fe(III) as Oxygen-Reduction Active Site**

Huogen Yu^a,b^,*, Jing Tian^b^, Feng Chen^b^*, Ping Wang^b^, Xuefei Wang^b^

^a^ State Key Laboratory of Silicate Materials for Architectures, Wuhan University of Technology, Wuhan 430070, People’s Republic of China

^b^ School of Chemistry, Chemical Engineering and Life Sciences, Wuhan University of Technology, Wuhan 430070, People’s Republic of China

*****Corresponding authors

Phone: 0086-27-87871029. Fax: 0086-27-87879468.

E-mail: yuhuogen@whut.edu.cn (H.Yu); fchen@whut.edu.cn

**Supporting Information**

Figure S1. XRD patterns of (a) TiO_2_; (b) rGO-TiO_2_; (c) Fe(III)/TiO_2_; (d) Fe(III)/rGO-TiO_2_; inset showing the XRD patterns of GO and rGO.

It is clear that the GO usually shows a characteristic diffraction peak at 2θ = 11.0 (inset in [Figure](http://www.sciencedirect.com/science/article/pii/S0926337312005772#fig0015) S1). After hydrothermal treatment of the GO nanosheets, a new and wide diffraction peak (ca. 24.1°) belonging to rGO is clearly observed owing to the reduction of GO (inset in [Figure](http://www.sciencedirect.com/science/article/pii/S0926337312005772#fig0015) S1), indicating that the hydrothermal treatment is an effective method for the reduction of GO to rGO. However, no diffraction peak of rGO can be observed in the rGO-TiO_2_ and Fe(III)/rGO-TiO_2_ composites (Figure 3b and 3d) due to its limited amount.

Figure S2. The rate constant (k) of photocatalytic decomposition of MO aqueous solution for various amounts of Fe(III) cocatalyst in the Fe(III)/rGO-TiO_2_ composites.

Figure S3. The repeated decomposition of DMP for the Fe(III)/rGO-TiO_2_ photocatalyst

**
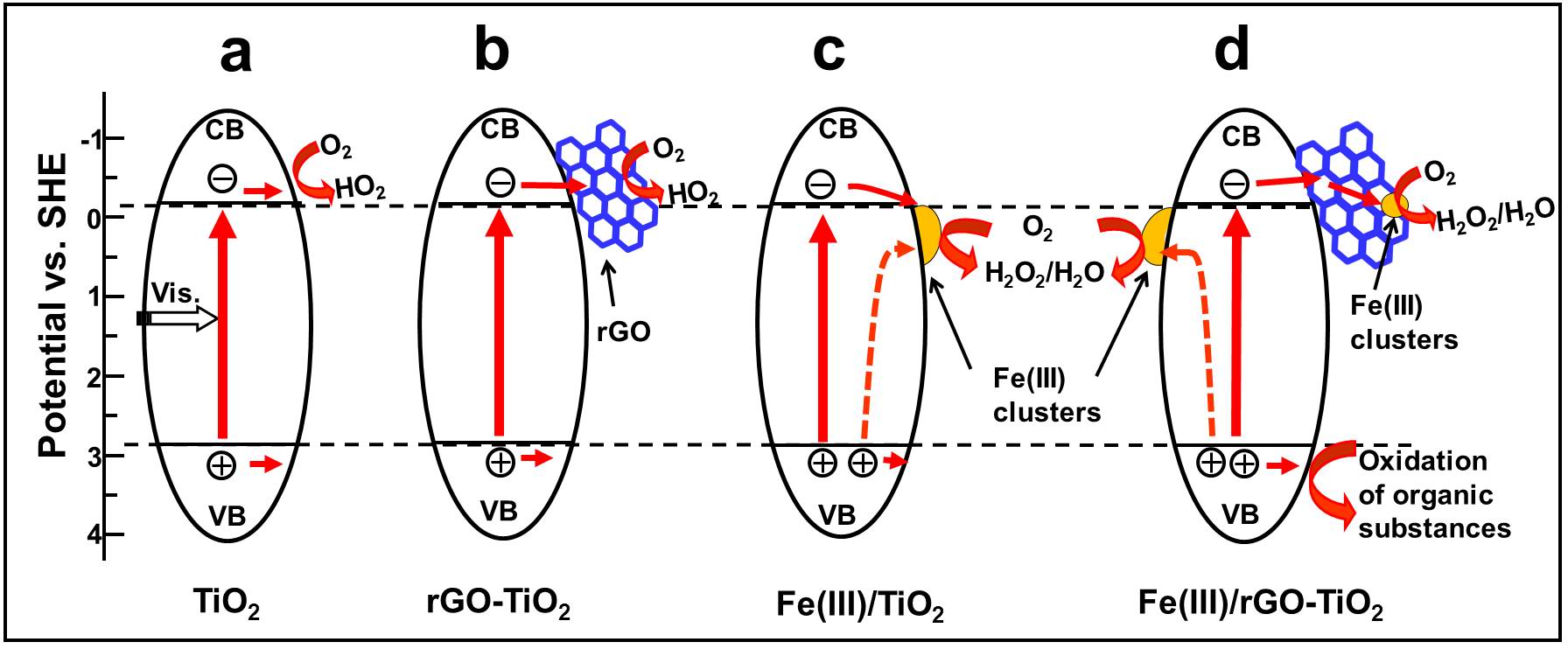
**

Figure S4. Schematic diagrams illustrating the possible photocatalytic mechanism of the (a) TiO_2_; (b) rGO-TiO_2_; (c) Fe(III)/TiO_2_; (d) Fe(III)/rGO-TiO_2_.

Figure S5 The UV-light photocatalytic performance of P25, rGO-P25 and Fe(III)/rGO-P25.
